# Genetic association analyses of atopic illness and proinflammatory cytokine genes with type 1 diabetes

**DOI:** 10.1002/dmrr.1259

**Published:** 2011-11-08

**Authors:** Nada M Saleh, Srilakshmi M Raj, Deborah J Smyth, Chris Wallace, Joanna M M Howson, Louise Bell, Neil M Walker, Helen E Stevens, John A Todd

**Affiliations:** Juvenile Diabetes Research Foundation/Wellcome Trust Diabetes and Inflammation Laboratory, Department of Medical Genetics, Cambridge Institute for Medical Research, University of Cambridge, Addenbrooke's HospitalCambridge CB2 0XY, UK

**Keywords:** autoimmune diabetes, atopy, asthma, cytokines, proinflammation

## Abstract

**Background:**

The genetic basis of the autoimmune disease type 1 diabetes (T1D) has now been largely determined, so now we can compare these findings with emerging genetic knowledge of disorders and phenotypes that have been negatively or positively associated with T1D historically. Here, we assessed the role in T1D of variants previously reported to be associated with atopic diseases and epithelial barrier function, profilaggrin (), and those that affect the expression levels of the proinflammatory cytokines tumour necrosis factor (TNF)-α, interleukin (IL)-1β, interferon (IFN)γ and IL-18.

**Methods:**

We genotyped single nucleotide polymorphisms (SNPs): − 105/rs28665122 in *SELS* or *SEPS1* (selenoprotein), three single nucleotide polymorphisms in *IL18* (−105/rs360717, + 183/rs5744292 and + 1467/rs574456) and R501X/rs61816761 in *FLG*, the major locus associated with atopic dermatitis and predisposing to asthma, in a minimum of 6743 T1D cases and 7864 controls.

**Results:**

No evidence of T1D association was found for any of the SNPs we genotyped at *FLG, SELS* or *IL18* (*p* ≥ 0.03), nor with haplotypes of *IL18* (*p* = 0.82). Review of previous T1D genome-wide association results revealed that four (human leucocyte antigen (HLA), gasdermin B/*ORM1 (Saccharomyces cerevisiae)*-like/gasdermin B/, *GSDMB*/*ORMDL3*/*GSDMA and IL2RB*) of ten loci recently reported to be associated with asthma were associated with T1D (*p*≤0.005).

**Conclusions:**

These results show that there are shared genetic associations for atopy-related traits and T1D, and this might help in the future to understand the mechanisms, pathways and environmental factors that underpin the rapid rise in incidence of both disorders in children. Copyright © 2011 John Wiley & Sons, Ltd.

## Introduction

Type 1 diabetes (T1D) is a multifactorial autoimmune disease which results from cell-mediated immune destruction of pancreatic β cells, a complex process controlled by many genes, in particular, the human leucocyte antigen (HLA) class II and class I genes 1. Considerable overlap in the map locations of susceptibility loci in T1D, celiac disease, Graves' disease and rheumatoid arthritis has been reported (http://www.t1dbase.org) indicating shared aetiologies 1, 2. However, T1D incidence has increased steadily over the last few decades, especially in children under 5 years of age, and is predicted to double again in this age group by 2020 3, 4. T1D shares this childhood pandemic with asthma, allergy and atopic illnesses, suggesting perhaps common environmental factors and susceptibility alleles 1, 3, 4. Atopy is the development of adverse hypersensitivity immune reactions against environmental antigens, usually associated with immunoglobulin E, and includes atopic dermatitis, asthma, allergic rhinitis, allergic conjunctivitis and food allergy 5. Stene and Joner found an inverse association between atopic dermatitis and T1D 6, supporting the EURODIAB substudy which found a reduced risk of T1D in children exhibiting atopic eczema 7. However, another group found that asthma was more common in children with T1D than those without in a Finnish birth register study, suggesting that Th1 and Th2 immune-mediated disorders can coexist 8.

Among the variants studied in atopic diseases, loss-of-function null mutations in the FLG gene are the most widely replicated and are the largest genetic risk factors for atopic dermatitis and also increase risk of asthma and peanut allergy 9–11. The two most common null *FLG* variants in the UK, R501X and 2282del4, are rare (<3%) and show high penetrance for atopic dermatitis in heterozygous and compound heterozygous genotypes. *FLG* encodes a precursor protein, profilaggrin, which is a constituent of the cornified envelope of the skin. Filaggrin plays a vital role in maintaining hydration levels in the epidermis and preventing the entry of potentially harmful chemical and biological antigens which can elicit immune responses. Therefore, *FLG* null mutations may increase skin permeability to molecules or pathogens which could influence T1D development.

Proinflammatory cytokines, such as TNF-α, IL-1β, IFN-γ, IL-1α, IL-6 and IL-18, have been implicated in the pathogenesis of T1D 12. Selenoprotein S (SELS) is involved in the retro-translocation of misfolded proteins from the endoplasmic reticulum to the cytosol in response to endoplasmic reticulum stress and inflammation, leading to the activation of transcription factor NF-κB, which in turn activates a number of genes including *IL1B, TNF* and *IL6*. Selenoprotein S is encoded by *SEPS1* (also known as *SEPS1* or *VIMP*), on chromosome 15q26.3 13. *SELS* has a single nucleotide polymorphism (SNP) in the promoter region located in a putative endoplasmic reticulum stress-response element. The minor A allele of SNP rs28665122 G > A is associated with decreased *SELS* expression and increased plasma levels of the proinflammatory cytokines IL-1β, TNF-α and IL-6 13.

IL-18 induces the production of IFNγ, TNF-α and IL-1. It has been implicated in the pathogenesis of several immune disorders including juvenile idiopathic arthritis and Crohn's disease 14, and serum IL-18 levels are higher in newly diagnosed T1D cases compared to controls 15. Recent studies have highlighted the importance of *IL18* haplotypic effects on *IL18* expression: *IL18* haplotypes carrying the C allele at − 105/rs360717 (5′ untranslated region) and G allele at + 183/rs5744292 (3′ untranslated region) are associated with a decrease in IL-18 at both the mRNA and protein level 16. Another allele associated with lower IL-18 serum levels is the C allele of the *IL18* intronic SNP rs5744256 (T > C), *p* = 1 × 10^−5^ 17. A recent genome-wide association study (GWAS) found two SNPs, rs2115763 and rs1834481, independently and convincingly associated with IL-18 levels 18. These SNPs are in linkage disequilibrium with rs5744256, *r*^2^ = 0.2, *D*′ = 1 and *r*^2^ = *D*′ = 1, respectively, in individuals of European ancestry in the Centre D'Etude du Polymorphisme Humain DNA samples. However, these SNPs have yet to be directly tested for T1D association. The recent asthma GWAS identified several regions including an association within the *IL18R1* gene 19.

In this study, we investigated whether SNPs in *SELS, IL18* and *FLG* were associated with T1D. We also investigated whether there was any overlap between the genetic regions associated with asthma 19 and T1D 20, 21.

## Methods

### Subjects and genotyping

Genotyping was performed on DNA samples from a minimum of 6743 British childhood-onset T1D cases and 7864 British controls, all of whom were of self-reported white ethnicity 22. Samples were genotyped for rs28665122, rs360717, rs5744292, rs5744256 and rs61816761 using TaqMan® allele discrimination assays developed by Assay-By-Design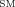 (Applied Biosystems, Warrington, UK), following the manufacturer's protocol, as described previously 22. The appropriate ethics committees approved the collection of all DNA samples, and written consent was obtained from all individuals, or parents of individuals who were too young to consent.

### Candidate gene association tests

Statistical analyses were performed using STATA version 10 (http://www.stata.com). Association was tested, and odds ratios with 95% confidence intervals calculated, by logistic regression models. Disease status was treated as the outcome variable in the logistic model and the allele/genotype of the test SNP used as independent variable(s). Cases and controls were stratified within the regression models into 12 broad regions of Great Britain to account for variation in disease incidence and allele/genotype frequency across the country 23, 24. The appropriateness of the multiplicative allelic effects model assumption was tested using a likelihood ratio test. Haplotypes were generated using SNPHAP (www-gene.cimr.cam.ac.uk/clayton/software) and tested for association using a logistic regression model. Disease status was used as the dependant variable and counts of haplotypes weighed by the posterior probabilities as independent variables. Geographical region was included as strata. Robust variance estimates were used and a multiplicative effects model assumed. Age-at-diagnosis effects were tested in cases, in a linear regression model with age-at-diagnosis as dependent variable and SNP genotype as independent variable.

We had > 90% power to detect an effect with an odds ratio ≥ 1.12 for an α of 10^−7^ for rs28665122 (*SELS*) and > 80% power to detect a similar effect for the *IL18* SNPs. *FLG* null alleles are rare (frequency < 3%), and, hence even with our large sample size the study was underpowered.

## Results

We genotyped the most common *FLG* null mutation R501X/rs61816761 in 7688 T1D cases and 9354 controls but no association was found (*p* = 0.82; Table 1). As the effect of null alleles in *FLG* is semi-dominant, in addition to genotyping R501X/rs6181671, we genotyped the second most common null mutation in this gene, 2282del4, which has a reported minor allele frequency (MAF) of 0.01–0.02 9 in 384 individuals (a random selection of cases and controls), and only one heterozygous individual was identified. Owing to the low MAF of these *FLG* null mutations and their semi-dominant mode of inheritance, over 11,000 cases and the same number of controls would be required to have 80% power to detect an association with an odds ratio ≥ 1.2 between *FLG* variants and T1D at α = 0.05. Therefore, no further samples were genotyped.

**Table 1:** Association of *FLG* single nucleotide polymorphism, R501X/rs61816761 (C > T), in 7688 type 1 diabetic cases and 9354 controls

Minor allele/genotype	Controls *n* (frequency)	Cases *n* (frequency)	Odds ratio (95% confidence interval)	*p*-value
T	452 (0.02)	368 (0.02)	1.01 (0.88–1.17)	0.82
C/C	8907 (0.95)	7323 (0.95)	1.00 (reference)	—
T/C	442 (0.05)	362 (0.05)	1.02 (0.88–1.18)	—
T/T	5 (0.00)	3 (0.00)	0.88 (0.20–3.81)	—

The *p*-value reported is for the multiplicative allelic effects model (which has an appropriate assumption).

*n*, number of chromosomes with allele T or number of individuals carrying the listed genotype.

The SNP -105/rs28665122 within *SELS* is the most associated SNP with proinflammatory cytokine levels 13, but was not associated with T1D risk (*p* = 0.08; Table 2). The three SNPs associated with IL-18 levels, − 105/rs5744292, + 1467/rs5744256 and + 183/rs360717, were also not associated with T1D (*p* ≥ 0.03; Table 3), nor were the *IL18* haplotypes defined by the two SNPs − 105/rs5744292 and + 183/rs360717 (*p* = 0.82; Table 4), which have been shown to be associated with decreased IL-18 levels 16, 25.

**Table 2:** Association of *SELS* single nucleotide polymorphism, -105/rs28665122 (G > A), in 8063 type 1 diabetic cases and 10,320 controls

Allele or genotype	Controls *n* (frequency)	Cases *n* (frequency)	Odds ratio (95% confidence interval)	*p*-value
A	2715 (0.17)	2020 (0.15)	0.94 (0.88–1.01)	0.08
G/G	7792 (0.76)	6160 (0.76)	1.00 (reference)	—
G/A	2341 (0.23)	1786 (0.22)	1.02 (0.89–1.18)	—
A/A	187 (0.02)	117 (0.02)	0.89 (0.21–1.26)	—

The *p*-value reported is for the multiplicative allelic effects model (which fitted the data).

*n*, number of chromosomes with allele A or number of individuals carrying the listed genotype.

**Table 3:** Association of *IL18* variants with type 1 diabetes

	*n*			*n* (frequency)			
					
SNP	Controls	Cases	Minor allele	Controls	Cases	Odds ratio (95%	*p*-value
			genotype			confidence interval)	
− 105/rs5744292	7864	6743	C	3932 (0.25)	3405 (0.25)	1.01 (0.96–1.07)	0.60
			T/T	4392 (0.56)	3765 (0.56)	1.00 (reference)	—
			T/C	3012 (0.38)	2551 (0.38)	0.99 (0.92–1.06)	—
			C/C	460 (0.06)	427 (0.06)	1.09 (0.94–1.25)	—
+ 1467/rs5744256	7904	6755	C	4112 (0.26)	3581 (0.27)	1.02 (0.97–1.08)	0.39
			T/T	4313 (0.55)	3667 (0.54)	1.00 (reference)	—
			T/C	3070 (0.39)	2595 (0.38)	0.99 (0.93–1.06)	—
			C/C	521 (0.07)	493 (0.07)	1.11 (0.97–1.27)	—
+ 183/rs360717	9093	7370	A	4900 (0.27)	3994 (0.27)	1.01 (0.96–1.06)	—
			G/G	4820 (0.53)	3945 (0.54)	1.00 (reference)	0.03
			G/A	3646 (0.40)	2856 (0.39)	0.95 (0.89–1.01)	—
			A/A	627 (0.07)	569 (0.08)	1.12 (0.99–1.26)	—

*p*-values are reported for the multiplicative allelic effects model at rs5744292 and rs5744256 as it was found to be an appropriate approximation, whereas the genotype effects model which makes no assumption about the mode of inheritance was required for rs360717.

**Table 4:** Association of the two SNP haplotypes, rs5744292 and rs360717, at *IL18* in 6123 type 1 diabetes cases and 7321 controls

		*n* (frequency)			
				
rs5744292	rs360717	Controls	Cases	Odds ratio (95% confidence interval)	*p*-value
T	G	7032 (0.48)	5804 (0.47)	1 (reference)	0.82
T	A	3946 (0.27)	3342 (0.27)	1.02 (0.96–1.09)	—
C	G	3635 (0.25)	3076 (0.25)	1.02 (0.96–1.09)	—

The C-A rs5744292-rs360717 haplotype had a frequency < 1% and hence is not listed.

The GABRIEL consortium asthma GWAS included 10 365 cases and 16 110 controls recruited from 23 different studies and identified nine regions (ten SNPs) associated with asthma, which are listed in Table S1. Among these regions, *ORLMD3*/*GSDMB* was the only non-HLA region shared between childhood-onset asthma and T1D (rs2305480 and rs3894194) 20. Review of the recent GWAS meta-analysis by Barrett *et al.* 20 revealed that the most atopy-associated SNPs in this region (as reported by several studies), rs2305480 and rs3894194, located in the introns of *GSDMB*, were also associated with T1D (*p* = 1.2 × 10^−6^, 2.7 × 10^−4^ and 9.3 × 10^−7^, respectively; Table S1). This was not unexpected as rs2305480 and rs7216389 are in high linkage disequilibrium with rs2290400, the most associated T1D SNP (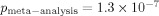) 20.

Although rs2284033, the asthma-associated SNP at *IL2RB*, showed suggestive evidence of association with T1D in the Barrett *et al.* meta-analysis 20 (*p* = 0.005; Table S1), other SNPs in the region were more convincingly associated with T1D (rs3218253, *p* = 2.54 × 10^−5^ 21; and rs229541, *p* = 1.98 × 10^−8^) 26). However, these T1D-associated SNPs are not in linkage disequilibrium with the asthma-associated SNP rs2284033 (*D*′ < 0.2) and, therefore, they probably have different effects on the expression of *IL2RB*, the strongest candidate gene in the region given the importance of the IL-2 pathway in T1D 1.

The GABRIEL consortium also showed that the SNP rs9273349 in the HLA class II region, close to *HLA-DRB1*, was associated with reduced risk of asthma (odds ratio = 0.85) 19. The SNP had not been typed by any of the GWAS used in the meta-analysis by Barrett *et al.* 20. However, rs1063355 near *HLA-DQB1* which is in linkage disequilibrium with rs9273349 (*r*^2^ = 1, *D*′ = 1 in the Centre D'Etude du Polymorphisme Humain DNA samples was included and so was used as a surrogate in the T1D study. The minor allele at these two SNPs (T) in *HLA-DQB1* confers protection in both asthma (odds ratio = 0.85) and T1D (odds ratio = 0.28; 20; Table S1). The minor T allele is in linkage disequilibrium with HLA-DQB1*06, HLA-DQB1*05, HLA-DRB1*15 and HLA-DRB1*01 (*r*^2^ = 0.48, 0.28, 0.27 and 0.19, respectively) which are known to confer reduced risk of T1D, specifically the HLA-DRB1*15-HLA-DQB1*06 haplotype (Table S2) 27. We tested for age-at-diagnosis effects at rs1063355 in 3977 T1D cases using the genotypes generated by Barrett *et al.* 20. The protective genotype at rs1063355 was found to be more common in relatively older onset T1D cases (*p* = 1.1 × 10^−4^) with an average age-at-diagnosis of 9.4 years for the cases homozygous for the protective (T) allele and 7.7 years for cases homozygous for the susceptibility allele.

## Discussion

We found no evidence for an obvious association between *IL18* SNPs and haplotypes and T1D susceptibility despite their known effects on IL-18 serum concentrations. This is consistent with a previous conclusion that the genes with functional variants associated with proinflammatory Th17 pathway-associated molecules and with immune diseases such as Crohn's disease do not alter T1D risk (e.g. *CCR6* and *IL23R*) 1, even though the role of proinflammatory cytokines, IL-1β, TNF-α and IL-17, in pancreatic β cell killing is established. Variants altering their levels are not risk factors for T1D, suggesting that cytokine-killing in T1D is a downstream consequence of the genetically determined autoimmune islet insulitis.

In contrast, one of the variants more associated with later-onset asthma, rs9273349, was located in the HLA class II region, which is the region in T1D with largest effect on risk. Alleles at this SNP show high linkage disequilibrium with several *HLA-DRB1-DQB1* haplotypes, but because the associations and the mechanisms underlying association of the HLA region with asthma are still not clearly defined, in contrast to T1D 27, a biological interpretation of this overlap cannot be made yet. Nevertheless, there is a significant link here, for the first time, between the diseases. Secondly, there is a link between diseases at the chromosome 17q12 and 17q21.1 loci, *GSDMB/ORMDL3/ GSDMA* (Table S1). The rs12936231 C allele is associated with higher risk of asthma and T1D and with higher expression of *GSDMB* and *ORMDL3* and lower expression of zona pellucida binding protein 2 *(ZPBP2)* 28. The biological consequences of this co-association remain to be determined.

The increase in the incidence of T1D and atopic illness in the past few decades in children also suggests that this increase is attributable to environmental or lifestyle factors as the genetic pool could not have changed enough to account for such an increase 3, 4. Exposure, or lack of exposure, to certain infectious agents and other homeostatic factors at an early age and the consequences that this could have on the development of the immune system has been an attractive explanation for the rise in incidence, which is supported by animal models of autoimmune disease 1, 5. However, the nature of the immunopathology underlying T1D and atopic illness involves different pathways and there is not much known about the role played by *GSDMB/ORMDL3/GSDMA, IL2RB* or HLA in atopic disease. Different gene–environmental interactions, with possible involvement of different microorganisms and dietary factors, including the composition of the gut microbiome with diverse effects on immune response and tolerance 29, undoubtedly operate in the aetiology of these two conditions.
