# Supplementary material for: Genetic association analyses of atopic illness and proinflammatory cytokine genes with type 1 diabetes
Source: Diabetes Metab Res Rev. 2011 Nov 8;27(8):838–43. doi: 10.1002/dmrr.1259 (PMC3816329; doi:10.1002/dmrr.1259)
Supplement: Supplementary file 1 [file dmrr0027-0838-SD1.doc]

**Supplementary Table 1 Association, with type 1 diabetes, of SNPs found to be significant (*P* < 5×10^-7^) in the GABRIEL consortium genome-wide asthma genetics study**

| Chr | Gene | SNP | MAF | | T1D | | Asthma | |
| --- | --- | --- | --- | --- | --- | --- | --- | --- |
|  |  |  | T1D Cases | Controls | OR (95% CI) | *P*-value | OR (95% CI) | *P*-value |
| 2 | *IL18R1* | rs3771166 G>A | 0.39 | 0.38 | 0.98 (0.94-1.02) | 0.25 | 0.87 (0.83-0.91) | 3.4 ×10^-9^ |
| 5 | *SLC22A5* | rs2073643 C>T | 0.43 | 0.45 | 1.06 (1.01-1.10) | 0.03 | 1.11 (1.06-1.15) | 2.2 ×10^-7^ |
| 5 | *IL13* | rs1295686 C>T | 0.18 | 0.18 | 1.01 (0.95-1.07) | 0.46 | 1.15 (1.09-1.20) | 1.4 ×10^-7^ |
| 6 | *HLA-DQB1* | rs1063355 G>T | 0.15 | 0.38 | 0.28 (0.26-0.30) | < 10^-230^ | 0.85 (0.81-0.88) ^ψ^ | 7.0 ×10^-14 ψ^ |
| 9 | *IL33* | rs1342326 A>C | 0.16 | 0.16 | 1.00 (0.96-1.05) | 0.96 | 1.20 (1.13-1.28) | 9.2 ×10^-10^ |
| 15 | *SMAD3* | rs744910 A>G | 0.50 | 0.49 | 1.00 (0.96-1.05) | 0.75 | 1.12 (1.09-1.16) | 3.9 ×10^-9^ |
| 15 | *RORA* | rs11071559 C>T | 0.13 | 0.12 | 0.98 (0.92-1.05) | 0.57 | 0.85 (0.80-0.90) | 1.1 ×10^-7^ |
| 17 | *GSDMB* | rs2305480 G>A | 0.50 | 0.47 | 0.90 (0.86-0.94) | 1.2 ×10^-6*^ | 0.85 (0.81-0.90) | 9.6 ×10^-8^ |
| 17 | *GSDMA* | rs3894194 G>A | 0.43 | 0.44 | 1.07 (1.02-1.13) | 2.7 ×10^-4^ | 1.17 (1.11-1.23) | 4.6 ×10^-9^ |
| 22 | *IL2RB* | rs2284033 G>A | 0.42 | 0.44 | 1.07 (1.02-1.12) | 0.005 | 0.89 (0.86-0.93) | 1.2 ×10^-8^ |

*P-*values and OR are reported for the multiplicative allelic effects model (1 degree of freedom) using the major allele as reference. T1D *P*-values were taken from Barrett *et al.* ([www.t1dbase.org](http://www.t1dbase.org/)) and we have calculated ORs in T1D using the genotype data from Barrett *et al.* . ^ψ^ values reported for rs9273349, the most associated SNP for asthma in the HLA class II region, which is in perfect LD with rs1063355 (r^2^=*D’*=1) in CEPHs. *rs2305480 is the most associated SNP in asthma, however, it is not the most associated SNP in T1D. rs2290400 is the most associated T1D SNP in the region (*P* *=*1.3×10^-7^) and is in LD with rs2305480, r^2^=0.8. Chr, .chromosome; MAF, Minor allele frequency; OR, odds ratio for minor allele; CI, confidence interval.

**Supplementary Table 2 Frequencies of the *HLA-DRB1*-rs1063355-*HLA*-*DQB1*** haplotypes in 3,962 type 1 diabetes cases and 3,955 controls. Only haplotypes with frequencies > 1% are listed.

| ***HLA-DRB1*** | rs1063355 | *HLA-DQB1* | Haplotype frequency | |
| --- | --- | --- | --- | --- |
|  |  |  | Controls | Cases |
| 15 | T | 6 | 14.48% | 0.64% |
| 1 | T | 5 | 11.47% | 8.95% |
| 13 | T | 6 | 8.39% | 4.29% |
| 14 | T | 5 | 2.29% | 0.13% |
| 3 | G | 2 | 14.22% | 33.63% |
| 701 | G | 2 | 10.07% | 4.59% |
| 401 | G | 301 | 6.73% | 4.47% |
| 11 | G | 301 | 6.51% | 1.02% |
| 404 | G | 3 | 4.68% | 7.85% |
| 401 | G | 3 | 4.45% | 23.69% |
| 701 | G | 303 | 4.09% | 0.24% |
| 8 | G | 402 | 1.87% | 2.46% |
| 12 | G | 301 | 1.39% | 0.44% |
| 901 | G | 303 | 1.30% | 1.34% |
| 13 | G | 301 | 1.02% | 0.14% |
| 405 | G | 3 | 0.46% | 2.07% |
| 402 | G | 3 | 0.23% | 1.01% |

Haplotypes were generated separately for cases and controls using the haplo.stats library in R ([www.r-project.org](http://www.r-project.org/)).
